# An Optimization Method to Enhance the Accuracy of Noise Source Impedance Extraction Based on the Insertion Loss Method

**DOI:** 10.3390/mi16080864

**Published:** 2025-07-26

**Authors:** Rongxuan Zhang, Ziliang Zhang, Jun Zhan, Chunying Gong

**Affiliations:** College of Automation Engineering, Nanjing University of Aeronautics and Astronautics (NUAA), Nanjing 211106, China; rxzhang01@nuaa.edu.cn (R.Z.); zhangziliang@nuaa.edu.cn (Z.Z.); zhanjun@nuaa.edu.cn (J.Z.)

**Keywords:** electromagnetic interference (EMI), insertion loss method, sensitivity analysis, noise source impedance

## Abstract

The optimal design of electromagnetic interference (EMI) filters relies on accurate characterization of noise source impedance. The conventional insertion loss method involves integrating two distinct passive two-port networks between the linear impedance stabilization network (LISN) and the equipment under test (EUT). The utilization of the insertion loss to formulate a system of binary quadratic equations concerning the real and imaginary components of the impedance of the noise source enables the precise extraction of the magnitude and phase of the noise source impedance in theory. However, inherent inaccuracies in the insertion loss method during extraction can compromise impedance accuracy or even cause extraction failure. This work employs a series inductance method to overcome these limitations. Exact analytical expressions are derived for the magnitude and phase of the noise source impedance. Subsequently, the application scope of the series insertion loss method is analyzed, and the impact of insertion loss measurement error on noise source impedance extraction accuracy is quantified. Requirements for improving extraction accuracy are discussed, and method optimization strategies are proposed. The permissible range of insertion loss error ensuring a solution exists is deduced. Finally, simulation and experimental results validate the proposed approach in a buck converter.

## 1. Introduction

Advancements in wide-bandgap semiconductors, such as using silicon carbide and gallium nitride, enable converters to operate at higher switching frequencies, thereby diminishing the need for an isolation transformer within their primary circuits. This development facilitates filtering inductance and capacitance volume weight, which consequently enhances power density. However, this advancement concomitantly exacerbates the electromagnetic interference (EMI) problem of converters because of the high voltage transition rate (dv/dt) and current transition rate (di/dt) of switching devices [1]. To ensure that a converter complies with electromagnetic compatibility standards, EMI filters are installed at the converter input to suppress noise [2,3,4]. The filtering performance of both passive and active EMI filters is associated with noise source impedance [5,6]. In power electronic converter systems, noise source impedance predominantly exhibits a capacitive or inductive characteristic, which significantly varies with frequency. Consequently, the extraction of noise source impedance in power electronic converters is crucial for the optimal design of EMI filters.

In a switching mode power supply (SMPS), noise source impedance is composed of parasitic inductance and stray capacitance of internal components and wires. These elements are influenced by the characteristics of the internal components as well as the layout of the power supply and its installation. Moreover, the coupling relationship of these elements is complex and thus complicates the theoretical calculation of noise source impedance. Consequently, noise source impedance must be measured. The primary measurement methods for noise source impedance can be categorized as follows: active injection methods (e.g., voltage injection method), passive measurement techniques (e.g., insertion loss method, network analyzer method), and hybrid approaches (e.g., two-probe measurement). A brief comparative analysis of these methods is presented in Table 1 below.

A two-probe approach to noise source impedance extraction typically requires a vector network analyzer to generate a perturbation through a current probe and simultaneously measure the response [10,11,12,13,14,15]. In [10], the probe did not come into direct contact with the EUT, which eliminated the influence of the probe and cable. In [14], the LCR parameters of the noise source impedance were iteratively calculated using a self-organizing migrating algorithm based on the two-probe method. However, the coupling effects between probes have not been considered in the existing literature, and probe sensitivity affects the reliability of measurements [16]. Noise source impedance can also be determined through the measurement of insertion loss during the insertion of a known impedance or the introduction of a perturbation between an SMPS and LISN [8,17,18]. As shown in [8], the insertion of a known impedance can be used to calculate the magnitude of the noise source impedance. However, the exact value and phase cannot be determined. As outlined in [18], a system of equations was derived from the insertion loss that enables the extraction of the magnitude and phase of the noise source impedance. Theoretically, the accuracy of the measured insertion loss is directly proportional to the magnitude of the noise. Therefore, the extraction accuracy of these frequency points is enhanced. A significant advantage of this method is its simplicity and convenience for application. However, in actual implementation, a significant measurement error will lead to extraction failure. The previous literature has not provided a foundation for parameter values, nor has it analyzed the factors that contribute to the error. Because of these shortcomings, the method has limited effectiveness.

In the present study, we conducted an in-depth analysis of the problem. Section 2 employs the series insertion loss method for illustrative purposes and analyzes its underlying principle for noise source impedance extraction. The solution expression is derived, and the verification via simulation of method’s feasibility and validity is discussed. Section 3 deduces the allowable limits of insertion loss error based on the established expression. Additionally, we introduce the sensitivity of insertion loss measurement error to both the magnitude and phase of the noise source impedance. Factors and principles influencing this sensitivity are examined, along with requirements for optimizing extraction accuracy. Section 4 proposes an optimized method and procedure for noise source impedance extraction using the insertion loss method. Section 5 presents simulations and experiments demonstrating the extraction of common-mode (CM) noise source impedance from a Buck converter, thereby validating the research findings. Conclusions are presented in Section 6.

## 2. Principle of the Insertion Loss Method

**Figure 1:**
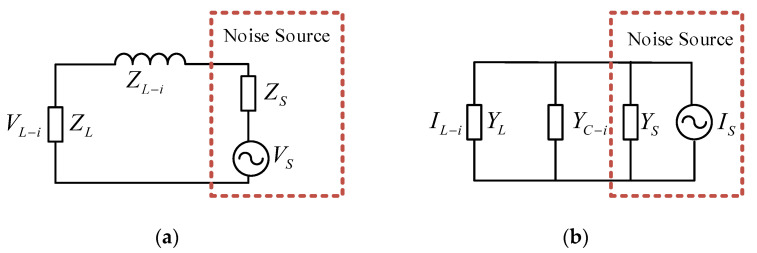
The equivalent circuits for extracting *Z_S_* using the (**a**) series inductance method and (**b**) parallel capacitance method.

The principle behind the insertion loss method for extracting noise source impedance is as follows: first, a known inductance or capacitance of different parameters is inserted twice between the EUT and LISN; then, the noise voltage in the frequency range of interest (i.e., 150 kHz–30 MHz) is measured using a spectrum analyzer or an EMI receiver. According to the insertion loss obtained from these two insertions, two sets of quadratic equations can be established for the real and imaginary parts of the noise source impedance. The magnitude and phase of the noise source impedance can then be calculated. The noise source impedance extraction equivalent circuits are shown in Figure 1.

Figure 1a presents the equivalent circuit when employing the series inductance method for noise source impedance measurement. *V_S_* denotes the noise source voltage, *Z_S_* signifies the noise source impedance, *Z_L_* represents the noise load impedance (and is generally equivalent to the LISN impedance), *Z_L-i_* indicates the inductance impedance of *ith* (*i* = 1, 2), and *V_L-i_* refers to the measured noise voltage at the *Z_L_* terminal. Figure 1b presents the equivalent circuit when using the parallel capacitance method for extracting the noise source conductance. *I_s_* denotes the noise source current, *Y_s_* signifies the noise source conductance, *Y_L_* represents the load conductance, *Y_C-i_* is the conductance of the inserted *ith* (*i* = 1, 2) parallel capacitor, and *I_L-i_* is the measured noise current. Assuming that *V_L_* and IL represent the noise voltage and current before insertion, respectively, insertion losses *IL_L-i_* and *IL_C-i_* of the series inductor and parallel capacitor can be calculated according to the definition of insertion loss.

(1) $$\left|IL_{L-i}\right|=20\mathrm{lg}\frac{\left|V_{L}\right|}{\left|V_{L-i}\right|}=20\mathrm{lg}\left|1+\frac{Z_{L-i}}{Z_{L}+Z_{s}}\right|$$

(2) $$\left|IL_{C-i}\right|=20\mathrm{lg}\frac{\left|I_{L}\right|}{\left|I_{L-i}\right|}=20\mathrm{lg}\left|1+\frac{Y_{C-i}}{Y_{L}+Y_{s}}\right|$$

As demonstrated in Equations (1) and (2), the expression of the parallel capacitance method is similar to that of the series inductance method. Therefore, we will analyze the series inductance method as an example.

The noise source impedance is denoted as *Z_S_* = *x_s_* + *jy_s_*, the known insertion inductance impedance is denoted as *Z_L-i_* = *a_i_* + *jb_i_*, and the load impedance is denoted as *Z_L_* = *a_L_* + *jb_L_*. According to Equation (1), a binary quadratic equation can be established to determine the real and imaginary parts of the noise source impedance.

(3) $$\left\{\begin{array}{c} \left|\frac{\left(x_{s}+a_{1}+a_{L}\right)+j\left(y_{s}+b_{1}+b_{L}\right)}{x_{s}+a_{L}+j\left(y_{s}+b_{L}\right)}\right|=10^{\frac{IL_{L-1}}{20}}=A_{1}\\ \left|\frac{\left(x_{s}+a_{2}+a_{L}\right)+j\left(y_{s}+b_{2}+b_{L}\right)}{x_{s}+a_{L}+j\left(y_{s}+b_{L}\right)}\right|=10^{\frac{IL_{L-2}}{20}}=A_{2} \end{array}\right.$$

In Equation (3), only the real part *x_s_* and the imaginary part *y_s_* of the impedance of the noise source are unknown, and hence the magnitude and phase of the *Z_S_* can be theoretically determined. According to [2], the two components of Equation (3) can be regarded as two circles, and the intersection of the two circles is the solution.

The coordinates of the center of the circle are:

(4) $$\left\{\begin{array}{c} x_{i}=-a_{L}+a_{i}/\left({A_{i}}^{2}-1\right)\\ y_{i}=-b_{L}+b_{i}/\left({A_{i}}^{2}-1\right) \end{array}\right.$$

The radius of the circle is defined as follows:

(5) $$r_{i}=\left[A_{i}\cdot \left|Z_{L-i}\right|\right]/\left({A_{i}}^{2}-1\right)$$

The horizontal and vertical coordinates of the intersection of the two circles can be found through the following equations:

(6) $$x_{s}=\frac{1}{2}\left(x_{1}+x_{2}\right)+\frac{r_{1}^{2}-r_{2}^{2}}{2D^{2}}\left(x_{2}-x_{1}\right)\pm \frac{1}{2}\sqrt{2\frac{r_{1}^{2}+r_{2}^{2}}{D^{2}}-\frac{{\left(r_{1}^{2}-r_{2}^{2}\right)}^{2}}{D^{4}}-1}\left(y_{2}-y_{1}\right)$$

(7) $$y_{s}=\frac{1}{2}\left(y_{1}+y_{2}\right)+\frac{r_{1}^{2}-r_{2}^{2}}{2D^{2}}\left(y_{2}-y_{1}\right)\pm \frac{1}{2}\sqrt{2\frac{r_{1}^{2}+r_{2}^{2}}{D^{2}}-\frac{{\left(r_{1}^{2}-r_{2}^{2}\right)}^{2}}{D^{4}}-1}\left(x_{2}-x_{1}\right)$$

Here, *D* is the distance of the circles and is derived as follows:

(8) $$D=\sqrt{{\left(x_{1}-x_{2}\right)}^{2}+{\left(y_{1}-y_{2}\right)}^{2}}$$

The magnitude of the noise source impedance is expressed as

(9) $$M=\sqrt{{x_{s}}^{2}+{y_{s}}^{2}}$$

Lastly, the phase of the noise source impedance is expressed as

(10) $$\varphi =\arctan\left(y_{s}/x_{s}\right)$$

In principle, two circles have two intersection points, and the intersection point with a positive *x_s_* is taken as the final result.

To verify the feasibility and accuracy of the above method, we conducted a simulation using the equivalent circuit shown in Figure 1a. The noise source impedance *Z_S_* was a 220 pF capacitor in series with a 20 Ω resistor; *L*_1_ was a 47 uH inductor with 6.5 kΩ parasitic resistance and 4.1 pF parasitic inductance; and *L*_2_ was a series of two connected *L*_1_ inductors.

**Figure 2:**
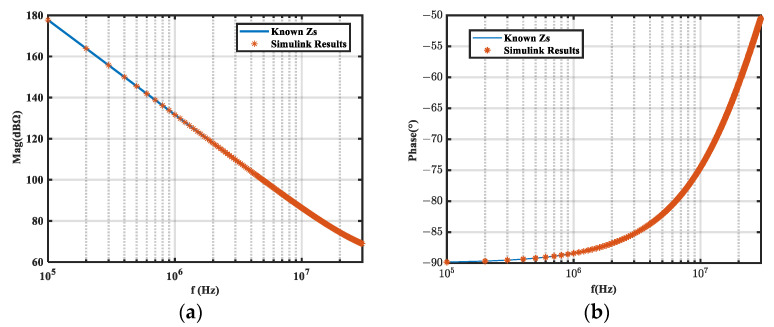
Extraction results of frequency domain simulation on equivalent circuits. (**a**) Magnitude; (**b**) phase.

The circuit was simulated in the frequency domain (100 kHz–30 MHz) with LTspice before and after the inductor was inserted. Insertion losses *A*_1_ and *A*_2_ were obtained under the two inductors, and the noise source impedance was calculated according to Equations (9) and (10). The simulation results were compared with the known noise source impedance, as shown in Figure 2. Since the insertion loss obtained by frequency sweeping was basically without error, the calculation results were very accurate. Hence, the feasibility and accuracy of the method were verified.

**Figure 3:**
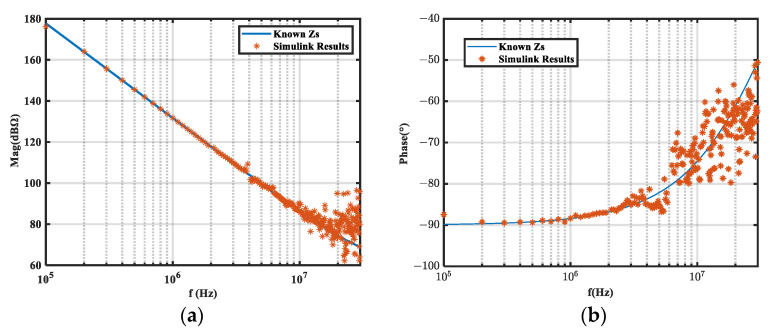
Extraction results of time domain simulation on equivalent circuits. (**a**) Magnitude; (**b**) phase.

**Figure 4:**
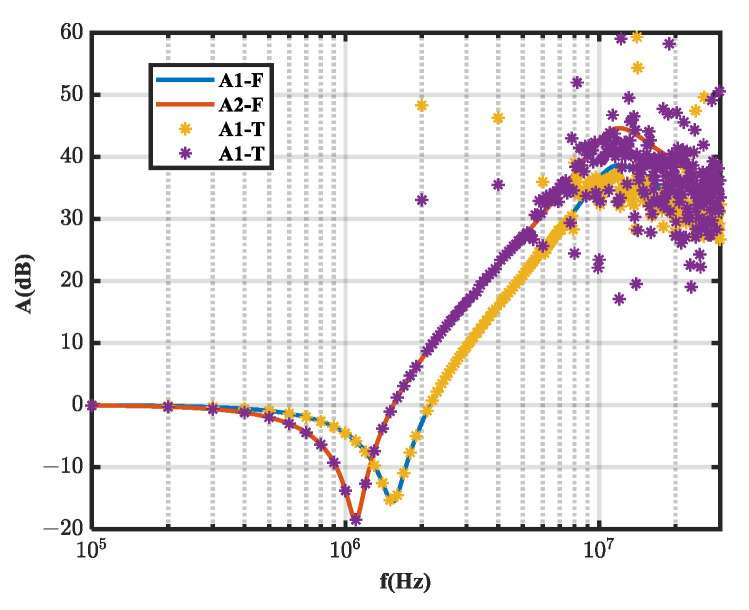
Comparison of insertion loss between time domain (*A*_1*-T*_, *A*_2*-T*_) and frequency domain simulations (*A*_1*-F*_, *A*_2*-F*_).

We performed a time domain simulation on the equivalent circuit with a 100 kHz square wave noise source. The load impedance voltages before and after inductor insertion were analyzed by FFT to obtain *A*_1_ and *A*_2_. The calculated noise source impedance was then compared to the known noise source impedance, as shown in Figure 3 and Figure 4, which compare the insertion loss obtained from the frequency domain (*A*_1*-F*_, *A*_2*-F*_) and time domain (*A*_1*-T*_, *A*_2*-T*_) simulations.

As shown in Figure 3 and Figure 4, the insertion loss (*A_i_*) error obtained from the time domain simulation caused noise source impedance extraction error.

## 3. Analyzing *A_i_* Error Impact on Extraction Results

As shown from the previous analyses, if the interpolation loss *A_i_* has no error, the exact noise source impedance can theoretically be extracted. However, in an actual test, the measurement value will inevitably have error due to instrumentation error, interference, and other reasons. For power electronic converters, which have strong interference, the measurement error of *A_i_* may be very large, which will inevitably cause subsequent extraction results to deviate or lead to extraction failing altogether. In this section, we analyze the rules dictating the effect of *A_i_* error on the extraction results. Through this, we aim to then optimize noise source impedance extraction and improve its accuracy.

To simplify our analysis, it is assumed that the series inductance has no parasitic parameter. Moreover, *a*_1_ = *a*_2_ = 0, *b*_1_ > 0, and *b*_2_ > 0; the measured values of *b*_1_ and *b*_2_ are assumed to be accurate. From the parametric equation of the trajectory circle, (*x*_1_ + *x*_2_)/2 = −*a_L_* is negative, and *x*_2_ − *x*_1_ = 0. Since the real part of the noise source impedance must be positive, the real part of the noise source impedance in Equation (6) and the imaginary part in Equation (7) can be simplified as follows:

(11) $$x_{s}=-a_{L}+\frac{1}{2}\sqrt{2\cdot \left(r_{1}^{2}+r_{2}^{2}\right)-\frac{{\left(r_{1}^{2}-r_{2}^{2}\right)}^{2}}{{\left(y_{2}-y_{1}\right)}^{2}}-{\left(y_{2}-y_{1}\right)}^{2}}$$

(12) $$y_{s}=\frac{1}{2}\left(y_{1}+y_{2}\right)+\frac{r_{1}^{2}-r_{2}^{2}}{2\left(y_{2}-y_{1}\right)}$$

Here,

(13) $$r_{1}=\left(A_{1}\cdot b_{1}\right)/\left({A_{1}}^{2}-1\right),r_{2}=\left(A_{2}\cdot b_{2}\right)/\left({A_{2}}^{2}-1\right),y_{1}=b_{1}/\left({A_{1}}^{2}-1\right),y_{2}=b_{2}/\left({A_{2}}^{2}-1\right)$$

The magnitude and phase of the noise source impedance are functions of *A*_1_, *A*_2_, *b*_1_, and *b*_2_. Furthermore, the theoretical relationship between *A*_1_, *A*_2_, *b*_1_, and *b*_2_ is

(14) $$\left\{\begin{array}{c} \left|\frac{\left(x_{s}+a_{L}\right)+j\left(y_{s}+b_{1}\right)}{x_{s}+a_{L}+j\left(y_{s}\right)}\right|=A_{1}\\ \left|\frac{\left(x_{s}+a_{L}\right)+j\left(y_{s}+b_{2}\right)}{x_{s}+a_{L}+j\left(y_{s}\right)}\right|=A_{2} \end{array}\right.$$

### 3.1. Constraints on the Solvable Equations

According to previous analysis, noise source impedance extraction is determined by the intersection of two trajectory circles. Therefore, to ensure that the system of equations has a solution, the two trajectory circles must have an intersection. Theoretically, if two different inductors are inserted, the two trajectory circles must intersect. In practice, however, if the measurement error of *A_i_* is too large, the two trajectory circles will not intersect and there will be no solution. The permissible range of the maximum deviation coefficient *K_i_* (*i* = 1, 2) when there is a solution is analyzed below.

**Figure 5:**
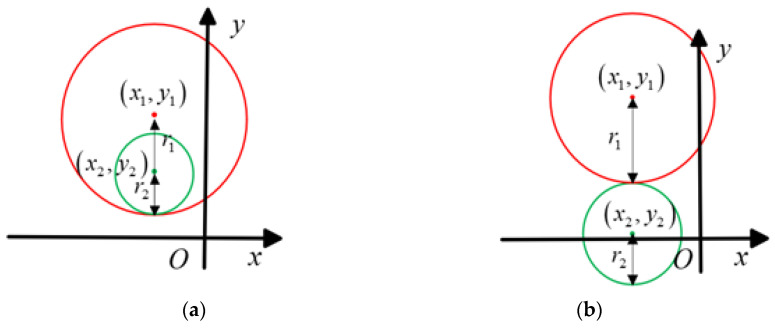
Schematic of the conditions necessary for trajectories to intersect. (**a**) Intratangent case; (**b**) extratangent case.

The necessary condition for the two trajectory circles to have an intersection point can be expressed by Equation (15). Figure 5 shows a schematic diagram of the tangency of two trajectory circles in cases where the system of equations has a solution.

(15) $$\begin{array}{l} r_{1}+r_{2}\geq \left|y_{1}-y_{2}\right|\\ \left|r_{1}-r_{2}\right|\leq \left|y_{1}-y_{2}\right| \end{array}$$

We assume that the insertion loss measurement is *A*_1_ · *K*_1_, where *A*_1_ is the theoretically accurate insertion loss, and *K*_1_ is the deviation coefficient. Additionally, we assume that *Z_S_* is much larger than *Z_L_* while *A*_2_ is much larger than *A*_1_.

Upon substituting Equation (13) into Equation (15), we obtain:

(16) $$\frac{A_{1}\cdot b_{1}}{\left({A_{1}}^{2}-1\right)}+\frac{\left(K_{2}\cdot A_{2}\right)\cdot b_{2}}{\left({\left(K_{2}\cdot A_{2}\right)}^{2}-1\right)}\geq \left|\frac{b_{1}}{\left({A_{1}}^{2}-1\right)}-\frac{b_{2}}{\left({\left(K_{2}\cdot A_{2}\right)}^{2}-1\right)}\right|$$

(17) $$\left|\frac{A_{1}\cdot b_{1}}{\left({A_{1}}^{2}-1\right)}-\frac{\left(K_{2}\cdot A_{2}\right)\cdot b_{2}}{\left({\left(K_{2}\cdot A_{2}\right)}^{2}-1\right)}\right|\leq \left|\frac{b_{1}}{\left({A_{1}}^{2}-1\right)}-\frac{b_{2}}{\left({\left(K_{2}\cdot A_{2}\right)}^{2}-1\right)}\right|$$

Pursuant to the aforementioned assumptions, the theoretical relationships between *b*_1_ and *b*_2_ and *A*_1_ and *A*_2_, as delineated in Equation (13), are substituted into Equations (16) and (17). Consequently, the range of the maximum deviation coefficient *K_i_* can be obtained.

(18) $$\left|\frac{\sqrt{{A_{1}}^{2}-1}-1}{A_{1}}\right|\leq K_{1}\leq \frac{1+\sqrt{{A_{1}}^{2}-1}}{A_{1}}$$

(19) $$\left|\frac{1-A_{1}}{\sqrt{{A_{1}}^{2}-1}}\right|\leq K_{2}\leq \frac{1+A_{1}}{\sqrt{{A_{1}}^{2}-1}}$$

**Figure 6:**
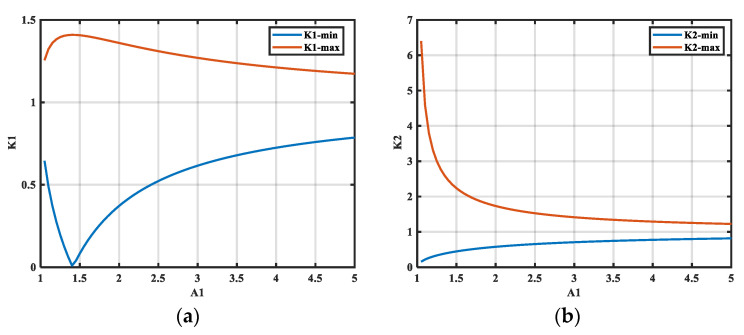
Range of the maximum deviation coefficient *K_i_* with insertion loss. (**a**) The range of *K*_1_; (**b**) the range of *K*_2_.

As demonstrated by Equations (18) and (19), the range of the deviation coefficient *K_i_*, which is predominantly associated with *A*_1_ and to a lesser extent with *A*_2_, is illustrated in Figure 6. The range of *K*_1_ increases initially and then decreases as *A*_1_ increases. The range of *K*_1_ is maximized when *A*_1_ *=* 1.414, and the range of *K*_2_ decreases with increasing *A*_1_.

**Figure 7:**
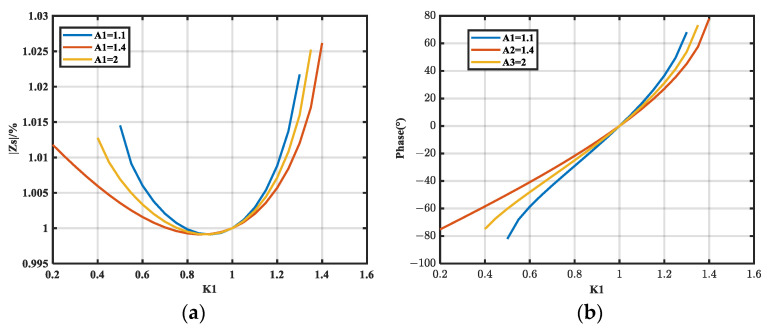
Impact of *K*_1_ on noise source impedance extraction. (**a**) Impact of *K*_1_ on magnitude accuracy; (**b**) impact of *K*_1_ on phase accuracy.

To verify the accuracy of the range of *K*_1_, the noise source impedance is taken to be a 1 kΩ resistor, and different inductors are inserted between the LISN and the simulated noise source. Assuming that *A*_2_ is tested accurately, *A*_2_ *=* 150. An artificial error, i.e., *K*_1_*·A*_1_, is added for *A*_1_, where *K*_1_ *=* 0.21.6. This is extracted according to the previously described method, and we then obtain the magnitude and phase accuracy, as shown in Figure 7.

As demonstrated in Figure 7, there is a discernible relationship between *K*_1_ and the increase in *A*_1_ for a specific value of *A*_2_; this corresponds to the observations shown in Figure 6a. Demonstrating maximum *K_i_* values between 0.36 and 1.36 when *A*_1_ = 2 and *A*_2_ = 150. The magnitude and phase accuracy of the noise source impedance initially increase and subsequently decrease with the rise in *A*_1_. Additionally, when *A*_1_ is close to 1.414, we observe the maximum *K*_1_ range with the highest extraction accuracy. Furthermore, the offset coefficient has a greater effect on the phase for the same *K*_1_. In the range extending beyond *K*_1_, the system of equations is not solvable, and the noise source impedance extraction is unsuccessful.

### 3.2. Effect of Insertion Loss Measurement Errors on Extraction Accuracy

#### 3.2.1. Sensitivity

To measure the effect of *A_i_* (*i* = 1, 2) error on impedance extraction accuracy, the relative value of the sensitivity of magnitude *M* to *A_i_* is introduced as follows:

(20) $$S_{i\text{-}M}=\left|\frac{dM\left(A_{1},A_{2},b_{1},b_{2}\right)}{dA_{i}}\cdot \frac{A_{i}}{\left|Z_{s}\right|}\right|$$

The relative value of the sensitivity of phase φ with respect to *A_i_* is given by

(21) $$S_{i\text{-}\varphi }=\left|\frac{d\varphi \left(A_{1},A_{2},b_{1},b_{2}\right)}{dA_{i}}\cdot A_{i}\right|$$

To simplify the following analysis, *A*_2_ is assumed to be much larger than *A*_1_ in the following analysis.

#### 3.2.2. Analyzing *S_i-M_* Under Resistive Noise Source Impedance

**Figure 8:**
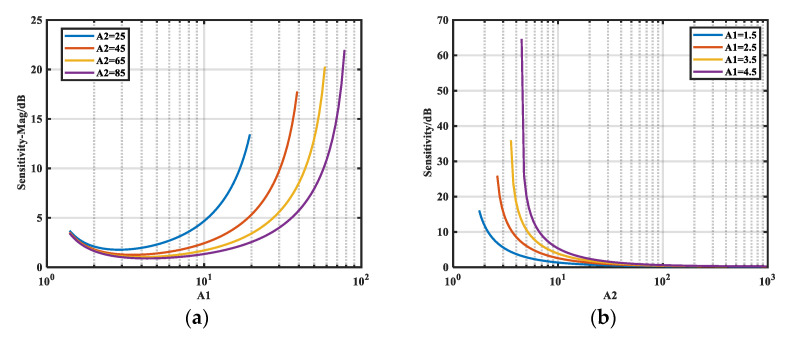
Trend of *S_i-M_* with *A_i_.* (**a**) Variation of *S*_1*-M*_ with *A*_1_ for different *A*_2_; (**b**) variation of *S*_2*-M*_ with *A*_2_ for different *A*_1_.

Here we use Equation (20) and the MATLAB R2018b software. The sensitivity of the magnitude of *Z_S_* to the relative error of *A*_1_ at 100 kHz is shown in Figure 8a, where *Z_S_* = 1 kΩ and *A*_2_ takes values of 25, 45, 65, and 85. Figure 8b presents the variation in sensitivity *S*_2*-M*_ of the magnitude of *Z_S_* to the relative error of *A*_2_ when *A*_1_ is 1.5, 2.5, 3.5, and 4.5.

As shown in Figure 8a, *S*_1*-M*_ decreases and then increases with increasing *A*_1_; moreover, it decreases as *A*_2_ increases. As shown in Figure 8b, *S*_2*-M*_ decreases with decreasing *A*_1_ but decreases with increasing *A*_2_. Finally, it converges to a constant lower limit of 0 dB, which can be derived theoretically from the following expression:

(22) $$S_{2\text{-}M\text{-}\min}=\left|\frac{dM}{dA_{2}}\cdot \frac{A_{2}}{\left|Z_{s}\right|}\right|\approx \left|1+\frac{Z_{L}}{Z_{s}}\right|$$

This expression is derived in Appendix A.

According to Equation (22), a lower limit exists for the sensitivity of the magnitude of *Z_S_* to the relative error of *A*_2_. Moreover, the minimal value of *S*_2*-M*_ is related to *Z_L_*/*Z_S_*. The larger the magnitude of *Z_S_*, the smaller the *S*_2*-M-*min_, and the lower limit is 1 (0 dB) when *Z_S_* is much larger than *Z_L_*. Thus, series insertion loss is suitable for the case where the noise source impedance magnitude is large.

#### 3.2.3. Analyzing *S_i-__φ_* Under Resistive Noise Source Impedance

**Figure 9:**
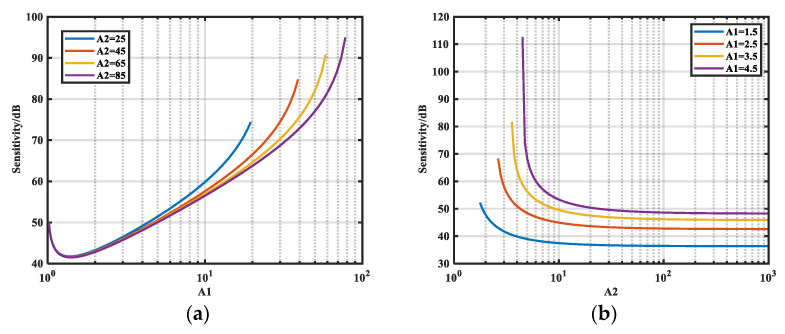
Trend of *S_i-__φ_* with *A_i_.* (**a**) Variation of *S*_1_*_-__φ_* with *A*_1_ for different *A*_2_; (**b**) variation of *S*_2_*_-__φ_* with *A*_2_ for different *A*_1_.

Under the same conditions as in Section 3.2.2, we analyze the variation in relative sensitivity *S_i-__φ_* of the phase of *Z_S_* to *A_i_*. The results are given in Figure 9.

Figure 9a shows the variation of *S*_1_*_-__φ_* with *A*_1_ for different *A*_2_. There exists a minimum sensitivity *S*_1_*_-__φ__-_*_min_ for *S*_1_*_-__φ_* when *A*_1_ is between 1 and 2. The variation of *S*_2_*_-__φ_* with *A*_1_ for different *A*_1_ is presented in Figure 9b. In this case, the sensitivity decreases with decreasing *A*_1_ for the same *A*_2_.

When A_2_ ≫ 1 and Z_S_ ≫ a_L_, *S*_2-_*_φ_* simplifies to:

(23) $$S_{2\text{-}\varphi }=\left|\frac{d\varphi }{dA_{2}}\cdot A_{2}\right|=\left|\frac{A_{2}\cdot \sqrt{A_{1}^{2}-1}}{\left(A_{2}-\sqrt{A_{1}^{2}-1}\right)}\right|$$

Equation (23) enables precise calculation of the relationship between A_1_ and A_2_ required for target sensitivity levels. Throughout this study, we maintained A_2_ ≥ 5A_1_, a readily satisfiable condition that generally ensures low sensitivity. Moreover, *S*_2*-*_*_φ_* decreases with increasing *A*_2_, and there exists a minimum limit *S*_2*-*_*_φ-_*_min_. The minimum limits *S*_1*-*_*_φ-_*_min_ and *S*_2*-*_*_φ-_*_min_ can also be derived theoretically:

(24) $$\begin{array}{c} S_{1\text{-}\varphi \text{-}\min}=\left|\frac{d\varphi }{dA_{1}}\cdot A_{1}\right|\approx \left|\frac{A_{1}^{2}}{\sqrt{A_{1}^{2}-1}}\right|\\ S_{2\text{-}\varphi \text{-}\min}=\left|\frac{d\varphi }{dA_{2}}\cdot A_{2}\right|\approx \left|\sqrt{A_{1}^{2}-1}\right| \end{array}$$

These expressions are derived in Appendix B.

#### 3.2.4. Analyzing *S_i-__φ_* and *S_i-M_* Under Capacitive Noise Source Impedance

**Figure 10:**
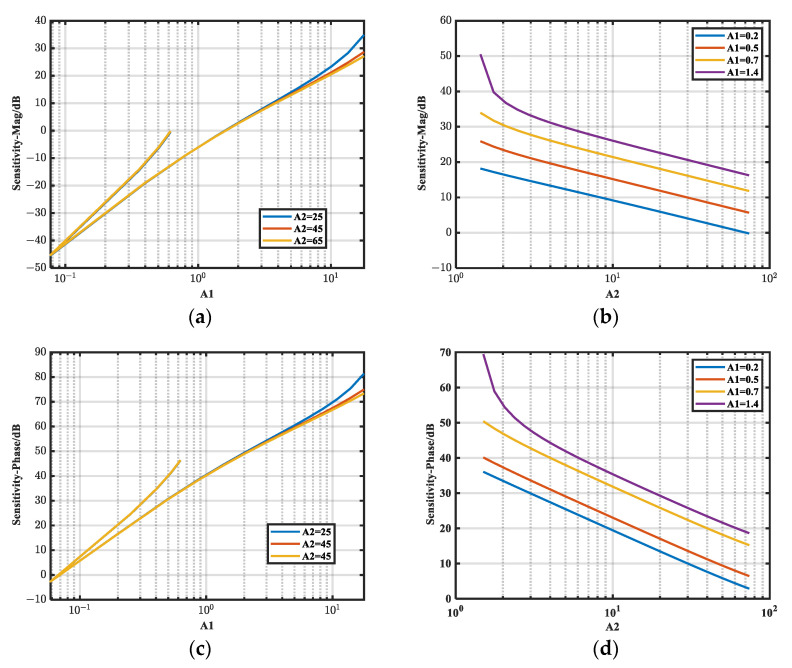
Trend of *S_ci-M_* and *S_c__i-__φ_* with *A_i_*. (**a**) Variation of *S_c_*_1*-M*_ with *A*_1_ for different *A*_2_; (**b**) variation of *S_c_*_2*-M*_ with *A*_2_ for different *A*_1_; (**c**) variation of *S_c_*_1_*_-__φ_* with *A*_1_ for different *A*_2_; (**d**) variation of *S_c_*_2_*_-__φ_* with *A*_2_ for different *A*_1_.

From the above analysis results under resistive noise source impedance, it can be seen that the assumed simulation and analysis results are credible. The same analysis method can be used to obtain the relative sensitivity relationship (*S_ci-M_*, *S_c__i-__φ_*) of the magnitude and phase under the capacitive noise source impedance. The results are presented in Figure 10.

As shown in Figure 10b,d, both *S_ci-M_* and *S_c__i-__φ_* decrease with an increasing *A*_2_*/A*_1_ ratio, which is consistent with the previous analysis. As observed in Figure 10a,c, the effect of *A*_2_ on *S_c_*_1*-M*_ and *S_c_*_1_*_-__φ_* is negligible when *A*_1_ is small. When *A*_1_ < 1.414, all sensitivities are smaller.

The same method can be used under the impedance of inductive noise, which is basically the same as capacitive impedance under the source sensitivity variation rule. The insertion loss would be constrained to the same value as the capacitive noise source impedance, but this is not analyzed in the present paper.

## 4. Optimization Methods to Improve Extraction Accuracy

The previous section analyzed the relative sensitivity rule of noise source impedance magnitude and phase to *A_i_*, and it derived the required *A_i_* value to obtain minimum sensitivity. It is evident that *A*_2_ is considerably larger than *A*_1_, and in practical applications *A*_2_ ≥ 5*A*_1_. When noise impedance is resistive, *A*_1_ = 1–3. For capacitive noise source impedance, *A*_1_ < 1.414. The conventional extraction method is fixed for two inductors, which complicates fulfillment of *A_i_* value requirements across a wide frequency range. Hence, the conventional method’s accuracy is only marginally superior in certain frequency ranges. Thus, we propose below an optimization method to ensure high accuracy across a wide range of frequencies.

(1) Test the noise spectrum of the device under test within the frequency band of interest and select several frequency points with relatively high noise amplitude within each decade of the frequency range.
(2) Select an inductor with any inductance value and insert it into the test to obtain the frequency characteristics of *A_i_* under that inductor. Then, determine the magnitude of the noise source impedance in different frequency bands. Determine the frequency band suitable for extracting the noise source impedance based on the requirements that A must meet.
(3) Based on the frequency band obtained in step 2, select *L*_1_ and *L*_2_ for the remaining frequency bands. Select inductors with higher inductance values for lower frequency bands and lower inductance values for higher frequency bands. Measure the impedance frequency characteristics of each inductor and the frequency characteristics of *A_i_* after insertion testing. Then, determine *L*_1_ and *L*_2_ for the different frequency bands based on the requirements.
(4) Substitute *A*_1_, *A*_2_, *Z_L-_*_1_, and *Z_L-_*_2_ into the analytical expressions for the magnitude and phase of the noise source impedance at each frequency point to calculate the impedance frequency characteristics of the noise source impedance.
(5) To organize the data, remove singularities to fit a relatively accurate noise source impedance curve. Note that it is critical to remove frequency points where the initial noise is relatively small, since these frequency points are subject to larger testing errors.

## 5. Simulation and Experimental Validation

### 5.1. Simulation Validation

**Figure 11:**
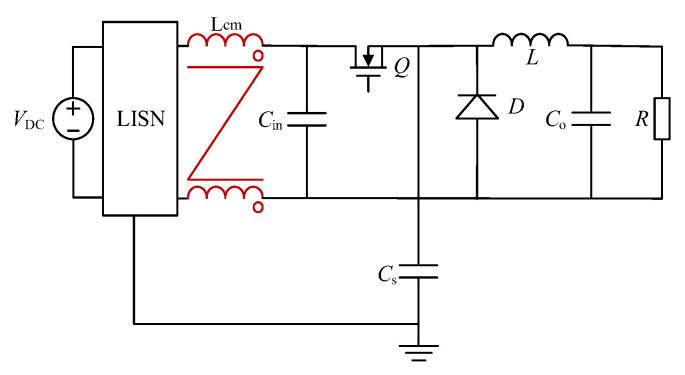
A typical buck converter circuit.

To verify the feasibility and effectiveness of the proposed optimization method, we employed the common mode noise source impedance *Z_S_* of a buck converter for simulations. The converter circuit is shown in Figure 11. The capacitor *C_S_* was equivalent to 220 pF *+* 20 Ω, and it was connected between the source of Q and the ground to represent *Z_S_*. In this configuration, the capacitor *C_S_* was significantly larger than the LISN equivalent impedance, which satisfied the application conditions for the previous iteration of the series inductance method. We utilized LTspice for simulations and used a simulation step of 10 ns. The parameters of the circuit are listed in Table 2.

**Figure 12:**
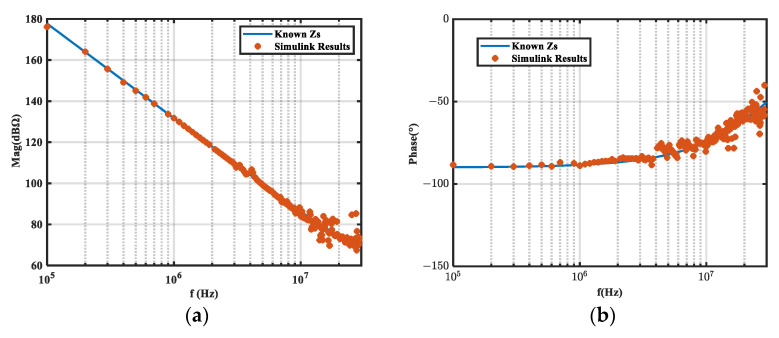
Extraction results of simulations on a BUCK converter. (**a**) Magnitude; (**b**) phase.

According to our proposed optimization method, various common mode inductors were connected, and the common mode insertion loss *A_i_* was obtained through simulation. By extracting the impedance of the actual converter shown in Figure 11, the relationships between the frequency range, inductance, and insertion loss were obtained and are given in Table 3. Figure 12 shows the extraction results.

**Figure 13:**
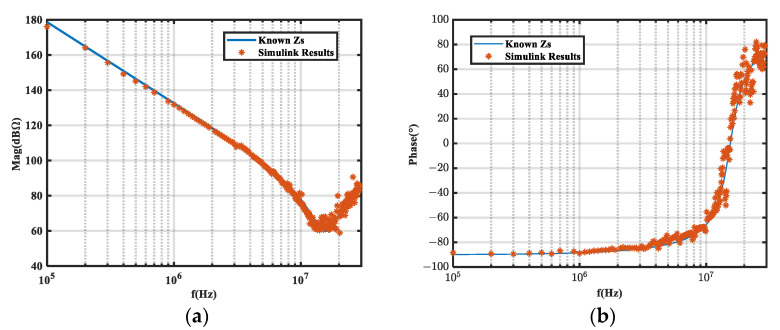
Extraction results of simulations on a BUCK converter with optimization method. (**a**) Magnitude; (**b**) phase.

The equivalent network of *C_s_* in Figure 11 was changed to an LCR network (220 pF + 500 nH + 20 Ω), and identical inductance values as shown in Table 2 were used for simulation validation. The results of extracting the noise source impedance are shown in Figure 13.

According to Figure 12 and Figure 13, when the noise source impedance is equivalent to different impedance networks and is much greater than the load impedance, *A*_1_ and *A*_2_ satisfy the value range of the optimization method within the frequency range of interest (i.e., 100 kHz to 30 MHz). In this case, the amplitude and phase of the impedance can be extracted with small errors. However, in the frequency band where the noise source impedance is not significantly greater than the load impedance (10 MHz to 20 MHz), the extraction results have relatively large errors.

**Figure 14:**
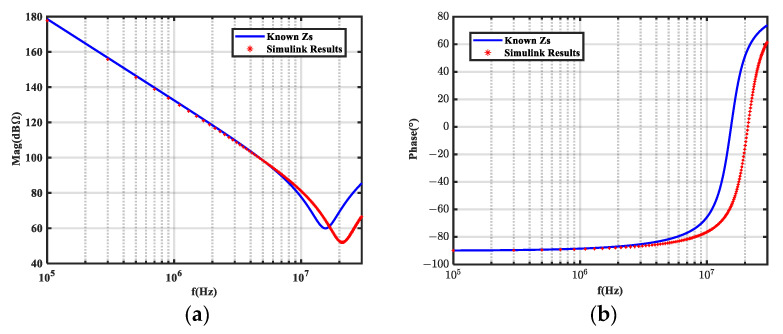
Extraction results of simulations on a BUCK converter with two-probe method. (**a**) Magnitude. (**b**) Phase.

The two-probe method, widely adopted for noise source impedance extraction, herein depended on [15], an S-parameter-based two-probe approach. The extraction results are presented in Figure 14.

As evidenced by the comparative analysis of Figure 13 and Figure 14, the two-probe method exhibited significant high-frequency errors. In Figure 14, despite employing relatively ideal current probe models in simulations, substantial high-frequency noise source impedance extraction errors persisted. Practical implementation limitations would further exacerbate these inaccuracies—not only via mutual coupling interference between probes but via bandwidth constraints inherent to physical probes. Furthermore, in contrast to our proposed methodology, the two-probe technique notably requires additional measurement instrumentation, specifically two precision high-frequency current probes and a vector network analyzer (VNA).

### 5.2. Experimental Validation

**Figure 15:**
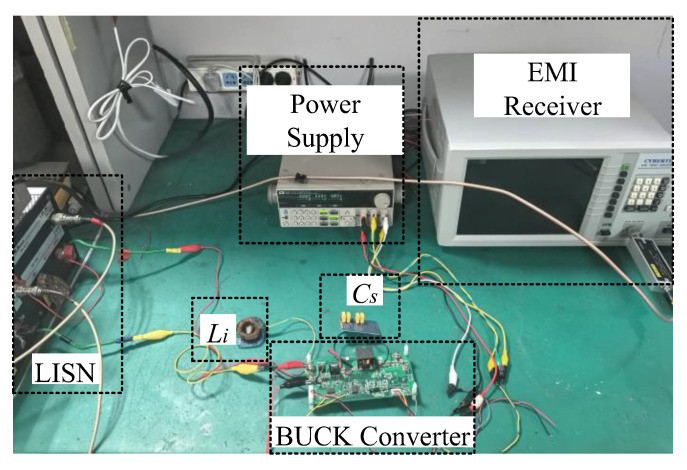
Practical setup for experimental validation using a buck converter.

**Figure 16:**
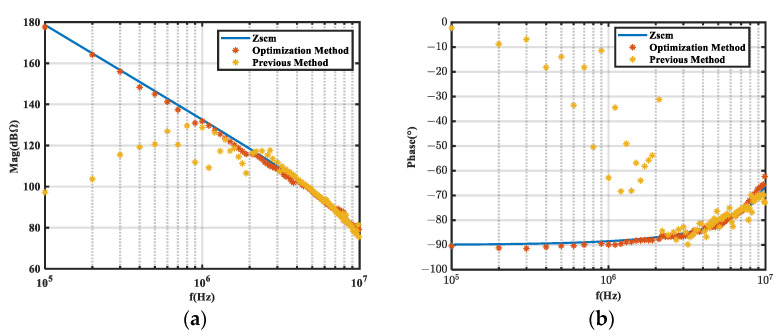
Experimental extraction results when using our optimization method with the optimization insertion loss method and the previous method. (**a**) Magnitude; (**b**) phase.

An experimental verification was performed using a buck converter with the same operating conditions as those given in Table 2. Since the parasitic capacitance *C_S_* was very small, a 220 pF CBB capacitor and a 20 Ω resistor were connected between the cathode of the BUCK converter diode and the heat sink to facilitate the comparison of the extraction accuracy as the noise source impedance *Z_scm_*. The ZLN3 vector network analyzer (Rohde&Schwarz, was sourced from Nanjing, China) was utilized to measure the impedance characteristics of the six inductors in Table 3 and those of the external *C_S_*. The insertion loss measured under different common mode inductors was measured with an EM5080B EMI receiver (Cybertek, was sourced from Nanjing, China). A photo of the experimental validation platform is given in Figure 15, while Figure 16 presents the experimental results.

As shown in Figure 16, the results confirmed that our optimized method achieved accurate noise source impedance extraction across the full frequency spectrum. In contrast, conventional IL methods exhibited substantial deviations or even failure due to their inability to satisfy the constraints on IL values analyzed before. The extracted CM noise source impedance was close to the actual impedance *Z_scm_* at 100 kHz to 10 MHz. Above 10 MHz, the main reasons for the decline in extraction results were as follows:

1. The noise generated by this buck converter was relatively low above 10 MHz. This made it difficult for the insertion loss to fully meet the specified value conditions, thereby affecting the extraction results.
2. At frequencies above 10 MHz, the noise source impedance was less than 75 Ω, making it difficult to fulfill the requirement for the noise source impedance to be significantly greater than the load impedance (25 Ω). This resulted in significantly increased sensitivity and a higher failure rate in the extraction results.

In subsequent analyses, we will test the noise source impedance in the high-frequency range using a parallel capacitor testing method.

## 6. Conclusions

The present paper analyzes the relationship between the impact of insertion loss measurement errors on the accuracy of the insertion loss method for extracting noise source impedance. We specifically employ a series inductance method, which is suitable for extracting the high noise source impedance. Our main contributions and conclusions are as follows.

(1) The causes of insertion loss measurement error were investigated, revealing that excessively large errors lead to solution failure. A permissible range and rule for insertion loss deviation ensuring solution existence are provided. When *A*_2_ is significantly larger than *A*_1_, the deviation coefficient ensuring a solution is found to depend primarily on the value of *A*_1_.
(2) The relative sensitivity of impedance magnitude and phase to insertion loss is analyzed. Analytical expressions for the limiting values of these relative sensitivities were theoretically deduced, and the relationship was established through simulation. Requirements for *A*_1_ and *A*_2_ are proposed to ensure higher extraction accuracy.
(3) Variation in the relative sensitivity of magnitude and phase to insertion loss under capacitive noise source impedance was examined using a proven simulation technique.
(4) An optimization method to enhance extraction accuracy is proposed. Its effectiveness was validated through simulations and experiments conducted on a buck converter.

## Figures and Tables

**Table 1:** A brief comparative analysis of different measurement methods.

Measurement Method	Principle	Advantages	Disadvantage
Voltage injection method [7]	Shunt-inject voltage V, measure current response I, Z = V/I.	Noninterruptive to main circuit; effective for low impedance.	Large errors at high impedance; bandwidth limitations of current probe.
Insertion loss method [8]	Insert precision inductor Z_L_, measure insertion loss A, Z ≈ |Z_L_|/A.	Low cost; no specialized equipment required.	Highly sensitive to insertion loss measurement error; unreliable when insertion loss is minimal.
Network analyzer method [9]	Measure S parameter, derive impedance Z.	Broad frequency coverage.	Complex setup requiring specialized probes/custom fixtures.
Two-probe method [10]	Measure ΔV with differential probes and current I with current probe, Z = ΔV/I.	Real-time dynamic response; nonintrusive; wide impedance measurement range.	High-frequency CMRR degradation; probe-induced errors; propagation delay matching; HF probe modeling challenges; notoriously complex calibration.

**Table 2:** Parameters of the BUCK converter.

Parameter	Value
V_DC_ (V)	30
Switching frequency (kHz)	100
Duty cycle	0.5
Output power (W)	5
*C*_s_ (pF)	220

**Table 3:** *L*_1_, *L*_2_, *A*_1_, and *A*_2_ for each frequency band.

Frequency Band (MHz)	*L*_1_ (μH)	*A*_1_	*L*_2_ (μH)	*A*_2_
0.1–0.3	1000	0.3–0.9	35,000	4–14
0.4–1	100	0.3–0.9	1000	1.2–7
1.1–4.1	10	0.3–0.8	1000	7.7–31
4.2–11	1	0.6–0.9	100	14–85
11.1–30	0.5	0.7–1.5	10	7–30

## Data Availability

Data is contained within the article.

## Appendix A

The following is the derivation of the expression of the lower limit of *S*_2*-M*_ at the resistive noise source impedance.

Assume that *A*_2_ is infinite. Since the impedance of the noise source is resistive, *y_s_* = 0. Thus,

(A1) $$y_{2}^{2}+{\left(x_{s}+a_{L}\right)}^{2}=r_{2}^{2}$$

By bringing Equation (13) into *y*_2_^2^/*r*_2_^2^, we obtain

(A2) $$\frac{y_{2}^{2}}{r_{2}^{2}}={\left(\frac{b_{2}}{{A_{2}}^{2}-1}/\frac{A_{2}\cdot b_{2}}{{A_{2}}^{2}-1}\right)}^{2}\approx {\left(\frac{1}{A_{2}}\right)}^{2}\approx 0$$

Then, by substituting (A2) into (A1), magnitude *M* can be obtained:

(A3) $$M=x_{s}\approx -a_{L}+r_{2}$$

By substituting (13) into (A3), we obtain

(A4) $$S_{2\text{-}M\text{-}\min}=\left|\frac{dM}{dA_{2}}\cdot \frac{A_{2}}{\left|Z_{s}\right|}\right|\approx \left|-\frac{b_{2}}{A_{2}\cdot \left|Z_{s}\right|}\right|$$

Given $A_{2}=\left|1+\frac{Z_{L2}}{Z_{L}+Z_{s}}\right|\approx \left|\frac{Z_{L2}}{Z_{L}+Z_{s}}\right|$, we then have

(A5) $$S_{2\text{-}M\text{-}\min}=\left|\frac{dM}{dA_{2}}\cdot \frac{A_{2}}{\left|Z_{s}\right|}\right|\approx \left|1+\frac{Z_{L}}{Z_{s}}\right|$$

## Appendix B

The following is the derivation of the expression of the lower limits of *S_i-__φ_* and *S*_2_*_-__φ_* at the resistive noise source impedance.

Since *Z_S_* is considerably larger than *Z_L_*, *x_s_* ≈ *Z_S_*. Thus, the phase expression can be approximated as follows:

(A6) $$\varphi =\arctan\left(y_{s}/\left|Z_{S}\right|\right)$$

After substituting (A6) and (12) into (21), we have

(A7) $$S_{2\text{-}\varphi }=\left|\frac{d\varphi }{dA_{2}}\cdot A_{2}\right|=\left|\frac{A_{2}^{2}\cdot \left(a_{L}+Z_{S}\right)\cdot \sqrt{A_{2}^{2}-1}\cdot \sqrt{A_{1}^{2}-1}}{Z_{S}\cdot \left(A_{2}^{2}-1\right)\cdot \left(\sqrt{A_{2}^{2}-1}-\sqrt{A_{1}^{2}-1}\right)}\right|$$

In the event that *A*_2_ is substantially larger than 1 and the noise source impedance *Z_S_* is significantly larger than *a_L_* (*Z_L_*), *S*_2_*_-__φ_* has a lower limit that can be obtained by

(A8) $$S_{2\text{-}\varphi \text{-}\min}=\left|\frac{d\varphi }{dA_{2}}\cdot A_{2}\right|\approx \left|\sqrt{A_{1}^{2}-1}\right|$$

Derivation of the *S*_1_*_-__φ_* expression is similarly possible:

(A9) $$S_{1\text{-}\varphi }=\left|\frac{d\varphi }{dA_{1}}\cdot A_{1}\right|=\left|\frac{A_{1}^{2}\cdot \left(a_{L}+Z_{S}\right)\cdot \sqrt{A_{2}^{2}-1}\cdot \sqrt{A_{1}^{2}-1}}{Z_{S}\cdot \left(A_{1}^{2}-1\right)\cdot \left(\sqrt{A_{2}^{2}-1}-\sqrt{A_{1}^{2}-1}\right)}\right|$$

*S*_1*-*_*_φ_* has a lower limit that can be obtained by

(A10) $$S_{1\text{-}\varphi \text{-}\min}=\left|\frac{d\varphi }{dA_{1}}\cdot A_{1}\right|\approx \left|\frac{A_{1}^{2}}{\sqrt{A_{1}^{2}-1}}\right|$$
